# AIDS prevention and control in the Yunnan region by T cell subset assessment

**DOI:** 10.1371/journal.pone.0214800

**Published:** 2019-04-18

**Authors:** Ya Li, Chenglu He, Zengpin He, Min Zhong, Dajin Liu, Ruiyang Liu, Ruixuan Fan, Yong Duan

**Affiliations:** 1 Yunnan Key Laboratory of Laboratory Medicine, Kunming, Yunan, China; 2 Yunnan Institute of Laboratory Diagnosis, Kunming, Yunan, China; 3 Department of Clinical Laboratory, The First Affiliated Hospital of Kunming Medical University, Kunming, Yunan, China; 4 Department of Medical Records and Statistics, The First Affiliated Hospital of Kunming Medical University, Kunming, Yunan, China; 5 Department of Standardized Training, The First Affiliated Hospital of Kunming Medical University, Kunming, Yunan, China; 6 Department of Infectious Diseases, The First Affiliated Hospital of Kunming Medical University, Kunming, Yunan, China; Fudan University, CHINA

## Abstract

**Background:**

Prior to being spread throughout broader China, multiple human immunodeficiency virus (HIV)-1 genotypes were originally discovered in the Yunnan Province. As the HIV-1 epidemic continues its spread in Yunnan, knowledge of the influence of gender, age, and ethnicity to instances of HIV reservoirs will benefit monitoring the spread of HIV.

**Methods:**

The degree to which T cells are depleted during an HIV infection depends on the levels of immune activation. T-cell subsets were assessed in newly-diagnosed HIV/AIDS patients in Yunnan, and the influence of age, gender, and ethnicity were investigated. Patients that were newly diagnosed with the HIV-infection between the years 2015 and 2018 at the First Affiliated Hospital of Kunming Medical College were selected for this study (N = 408). The lymphocyte levels and T cell subsets were retrospectively measured in whole blood samples by FACS analysis.

**Results:**

The median CD4 count was 224 ± 191 cells/μl. Significantly higher mean frequencies and absolute numbers were observed in CD3^+^, CD3^+^CD4^+^, CD3^+^CD8^+^, CD45^+^, and CD3^+^CD4^+^/CD45^+^ in females compared to males. Han patients showed a higher total number of CD3+T cells and the ratio of CD3^+^ /CD45^+^ cells compared to any other ethnic minority (P < 0.001). The numbers of CD3+ T-cells, CD3+CD8+ T cells, and CD45+ T cells were highest in the age group ≥ 60. Significant differences were observed in the counts of CD3+, CD3+CD8+, and CD45^+^ cells and the ratio of CD3^+^/CD45^+^ and CD3^+^CD4^+^/CD45^+^ cells between the ≤ 29 and 30–59 age groups.

**Conclusion:**

This study has revealed that low levels of CD4^+^ T cells can be observed in newly-diagnosed HIV/AIDS patients in the Yunnan province. It has also been demonstrated that gender, age, and ethnicity have a significant association with the ratio of T-cell subsets that may contribute to virus progression and disease prognosis in individuals belonging to certain subsets of the population. This study has highlighted the importance of HIV/AIDS screening in at-risk populations to ensure timely and adequate clinical management in Yunnan.

## Introduction

In the absence of antiretroviral therapy (ART), infection with HIV weakens the host’s immune system, leading to acquired immune deficiency syndrome (AIDS). The time between infection and clinical diagnosis of AIDS varies between individuals and can be influenced by age, gender, ethnicity, and sexual orientation [1].

AIDS is a serious public health problem in China [2–4]. Located in the Southwest region of China, the Yunnan province borders Vietnam, Myanmar, and Laos and contains a large scale of cross-border populations. Yunnan is also close to the Golden Triangle: the largest drug-producing area in China [5–7]. In 1989, the first HIV type-1 (HIV-1) outbreak occurred in injection drug users in the Dehong prefecture of Yunnan [8]. Since that outbreak, Yunnan has become the gateway for HIV-1 epidemics in China and is the most severely-affected HIV/AIDS area nationwide [9–11]. Recent statistics suggest that almost 25% of new HIV cases in China originate from Yunnan [12–14], 92.6% of which are caused by unprotected sex [9,15,16]. Epidemiological surveillance is vital to the control of this epidemic.

The ability of HIV to establish long-lasting viral reservoirs in CD4 T cells initiates rebound viremia during ART treatment [17–19]. This represents a distinct aspect of HIV-1 pathogenesis and a critical factor that contributes to the inability to cure HIV-1 infection [20]. Viral persistence is attributed to latently-infected CD4 T cells that contain transcriptionally silent forms of the virus that are not susceptible to ART drugs or immune factors (1). Traditionally, resting CD4 T memory cells are regarded as the major T cell population that harbor latent HIV-1, which become infected at the time of activation, and viral latency occurs when these cells revert to a resting condition [21–23].

CD4 T cell counts are the primary prognostic marker for HIV-related diseases and is the main indicator for ART therapy [20]. When CD4 T cell numbers decline below 200 cells/ul, the immune system is compromised, resulting in a poor disease prognosis and lack of response to ART therapy [12,20,24–27]. However, not all CD4 T cells are identical, and emerging data suggests a role for specific T-cell subsets during virus progression [23,28–30]. The ratio of these T cells in HIV patients can be influenced by age, gender, and ethnicity The various T-cell subpopulations likely play an important role in modulating not only HIV-specific immunity, but the overall immune response during infection [23,28–30].

The worldwide use of ART therapy is expanding [12,25–27]. With this increase, it is important to identify differences in immunological progression by gender, age, and ethnicity, as specific populations may require subtype-specific monitoring and guidelines for treatment [1,31]. Such estimates of CD4+ T cell trajectories are essential for public health models as they can predict the course of an HIV epidemic [20,24,32]. In this study, to enhance the understanding of T cell characteristics and disease progression in newly diagnosed HIV/AIDS patients in Yunnan, a comprehensive study was conducted that included patient samples collected across the entire province from 2015 to 2018. The aim of this study was to provide a meaningful reference for AIDS prevention and control strategies in Yunnan.

## Methods

### Ethics approval

The study protocol was reviewed and approved by the Ethics committee of the first affiliated hospital of Kunming Medical University. Informed consent was obtained from all participants prior to enrollment. Patient records and information were de-identified prior to analysis.

### Study population and detection of HIV infection

A retrospective study of HIV/AIDS cases in the Yunnan provinces was conducted. The survey subjects were newly diagnosed HIV-infected patients who had not received ART therapy. All patients were confirmed positive for HIV antibodies by standard laboratory detection methods, including Western blot analysis to detect HIV antibodies and nucleic acid as a supplementary test to detect the HIV load when necessary. All patient diagnoses were consistent with the national HIV/AIDS testing technical specifications.

### Blood samples

For newly diagnosed HIV-infected patients, 2 mL of venous blood samples were obtained from each patient to measure T cell subsets, with all analysis completed within 4 h of sampling. Flow cytometric acquisition and analysis was performed using a FACSCan II flow cytometer (BD Biosciences, San Jose, CA). Absolute numbers of each lymphocyte subset were determined using CD3/CD4/CD8/CD45 BD Multitest reagents according to the manufacturer’s protocols (BD Biosciences).

### Statistical analysis

SPSS22.0 software was used for statistical analyses. Continuous variables are presented as the mean ± Standard Deviation (SD). Two–two comparisons amongst the groups were performed using LSD methods. Two-tailed tests were used for statistical analysis. The threshold for statistical significance was a p-value < 0.05.

## Results

### Patient characteristics study

**Fig 1:**
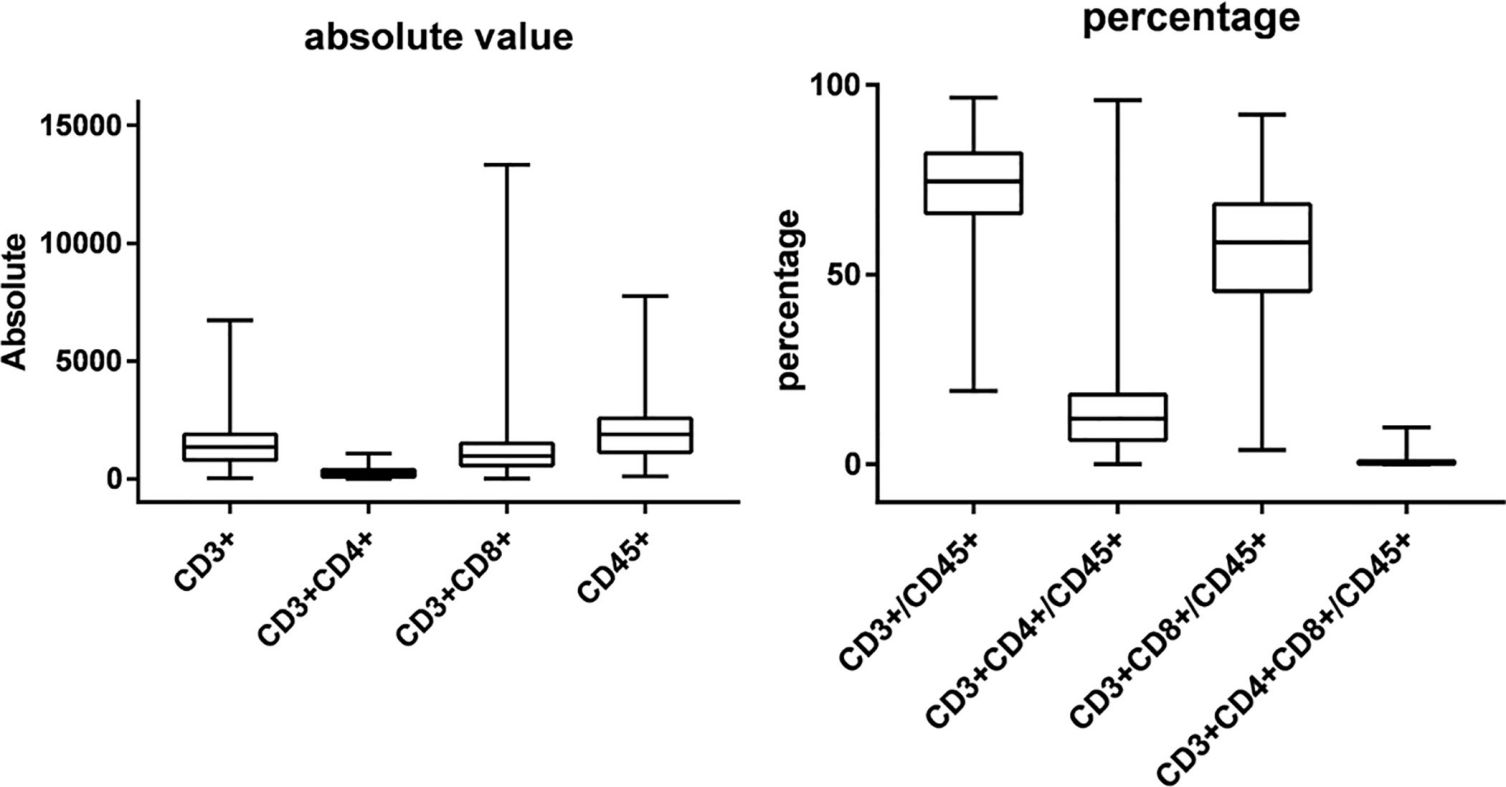
T lymphocyte subsets in newly diagnosed HIV/AIDS patients. The boxplots of CD3^+^, CD3^+^CD4^+^, CD3^+^CD8^+^, CD45^+^, CD3^+^/CD45^+^, CD3^+^CD4^+^/CD45^+^, CD3^+^CD8^+^/CD45^+^, and CD3^+^CD4^+^CD8^+^/CD45^+^.

A total of 408 newly diagnosed with HIV/AIDS patients were assessed. Table 1 and Table 2 summarize the basic characteristics of the study population. Fig 1 depicts the mean and standard deviation of CD3^+^, CD3^+^CD4^+^, CD3^+^CD8^+^, CD45^+^, CD3^+^/CD45^+^,CD3^+^CD4^+^/CD45^+^, CD3^+^CD8^+^/CD45^+^, and CD3^+^CD4^+^CD8^+^/CD45^+^ in two box plots. The HIV patients were grouped according to age (1) 30–59 years, (2) ≤ 29 years, and (3) ≥ 60 years. The study sample was primarily male (65%, n = 267), 11.8% were ethnic minorities, and the mean CD4 T cell count was 224 cells/μL across all individuals.

**Table 1:** Percentages of each ethnic group amongst newly diagnosed HIV/AIDS patients.

Ethnic group	Cases	Percentage(%)
Han nationality	349	85.5
Bai nationality	6	1.5
Bouyei nationality	4	1.0
Dai nationality	2	0.5
Hani nationality	7	1.7
Hui nationality	7	1.7
Lisu nationality	1	0.2
Miao nationality	1	0.2
Naxi nationality	3	0.7
Shui nationality	1	0.2
Yi nationality	19	4.7
Zhuang nationality	5	1.2
Pumi nationality	2	0.5
Blang nationality	1	0.2

**Table 2:** Distribution of newly diagnosed HIV/AIDS patients according to age.

Age group (years)	No. of new cases	Proportion of study population
≤29	80	19.6%
30–59	278	68.1%
≥60	50	12.3%

### T-lymphocyte subset analysis

**Fig 2:**
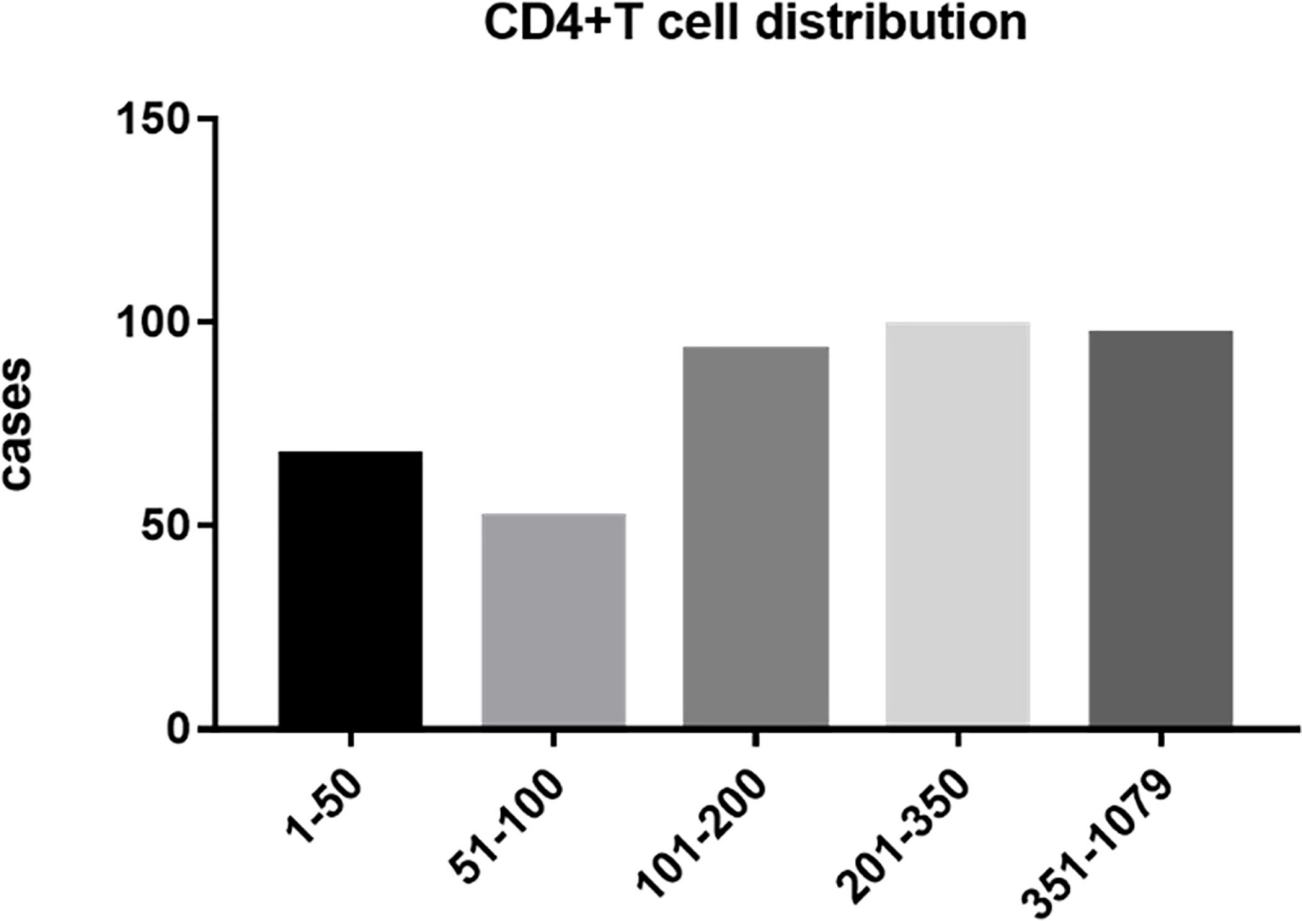
CD4+ T-cell counts in newly diagnosed HIV/AIDS patients. The bar graphs are presented by data of blood samples from newly diagnosed HIV-infected patients.

Baseline CD4 counts are a significant predictor of the progression of HIV-associated diseases [20]. The mean baseline CD3^+^CD4^+^ T cell counts of HIV-infected individuals in the study population were 224 cells/μl. The CD4 T-cell counts of the patients ranged from 1 to 1079 cells/μl. Of these individuals, 97 patients (23.8%) had baseline CD4 cell counts >350 cells/μl, while 99 patients (24.3%) had baseline CD4 cell counts ranging from 200–350 cells/μl. 93 patients (22.8%) had baseline CD4 cell counts 100–200 cells/μl, and 119 patients (29.1%) had CD4 T cell levels <100 cells/μl (Fig 2). The low levels of CD4 T cells in these patients suggested a diagnosis of an advanced stage of HIV infection [33].

### T cell subset frequencies in HIV+ men and women

**Fig 3:**
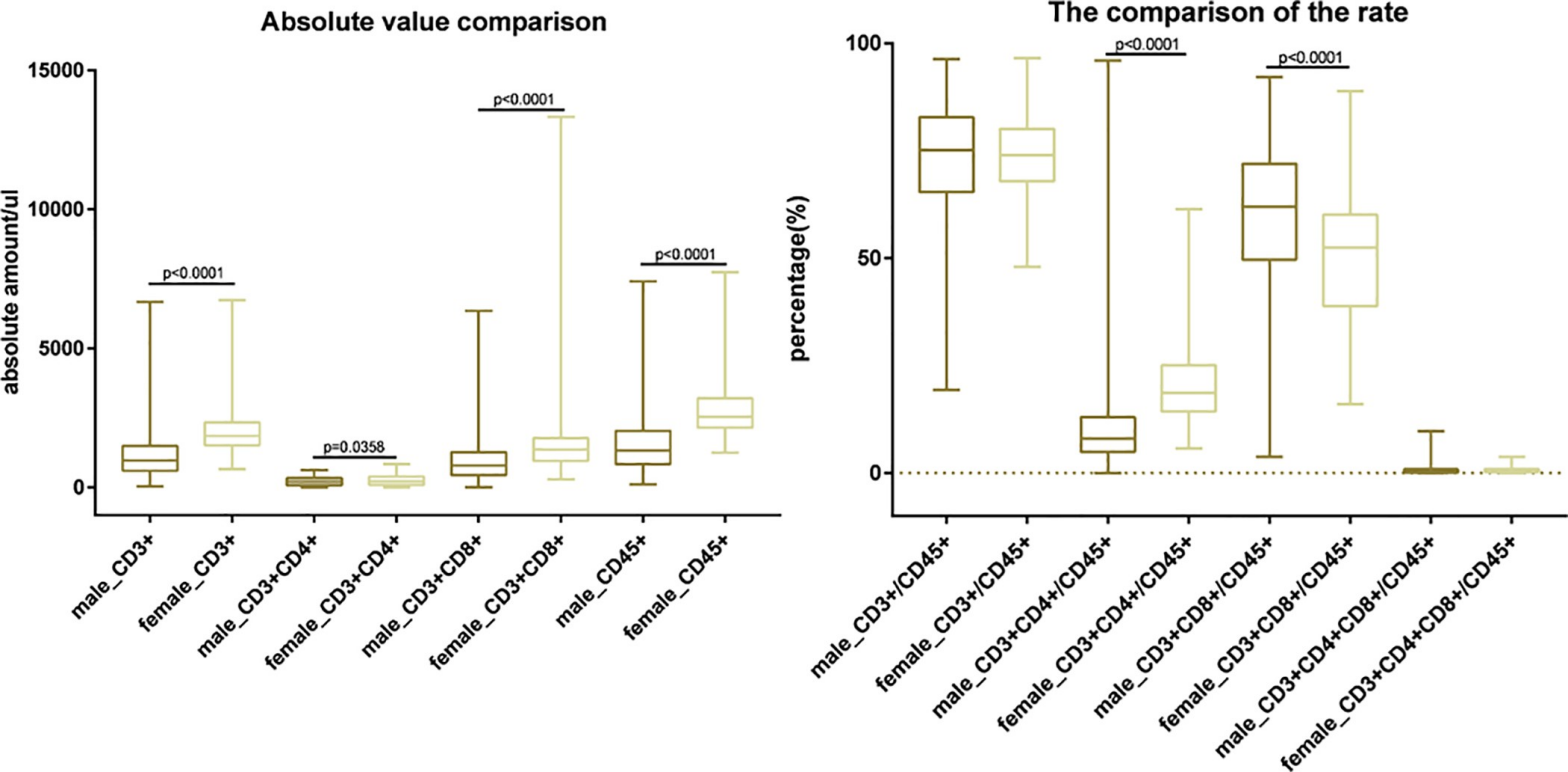
T lymphocyte subsets amongst men and women with newly diagnosed HIV/AIDS. FACs data are presented as the mean ± SD for the comparison of CD3/CD4/CD8/CD45 subsets between male and female patients.

Clinical studies indicate that female gender is associated with lower viral loads, higher CD4 counts, pronounced ART side effects, and a more rapid progression to AIDS, though this has not been shown in the Yunnan population [9,16,34–40]. When the mean absolute numbers of each T cell subset were assessed by gender (Fig 3), the mean percentage of CD3^+^CD8^+^/CD45^+^ T cells in men was higher than in women (P<0.001), while the mean frequencies and absolute numbers of CD3^+^, CD3^+^CD4^+^, CD3^+^CD8^+^, CD45^+^, and CD3^+^CD4^+^/CD45^+^ in female patients was higher than in male patients (P<0.01 or P<0.001), suggesting a functional skewing of the accompanying quantitative CD8 elevation. Since this would be predicted to fuel immune dysfunction in females infected with HIV in Yunnan [41], a combination of immunological strategies to supplement ART may be required to recover CD8 T-cell normalization and improve HIV symptoms in these patients.

### T lymphocyte subset frequencies and ethnicity

**Fig 4:**
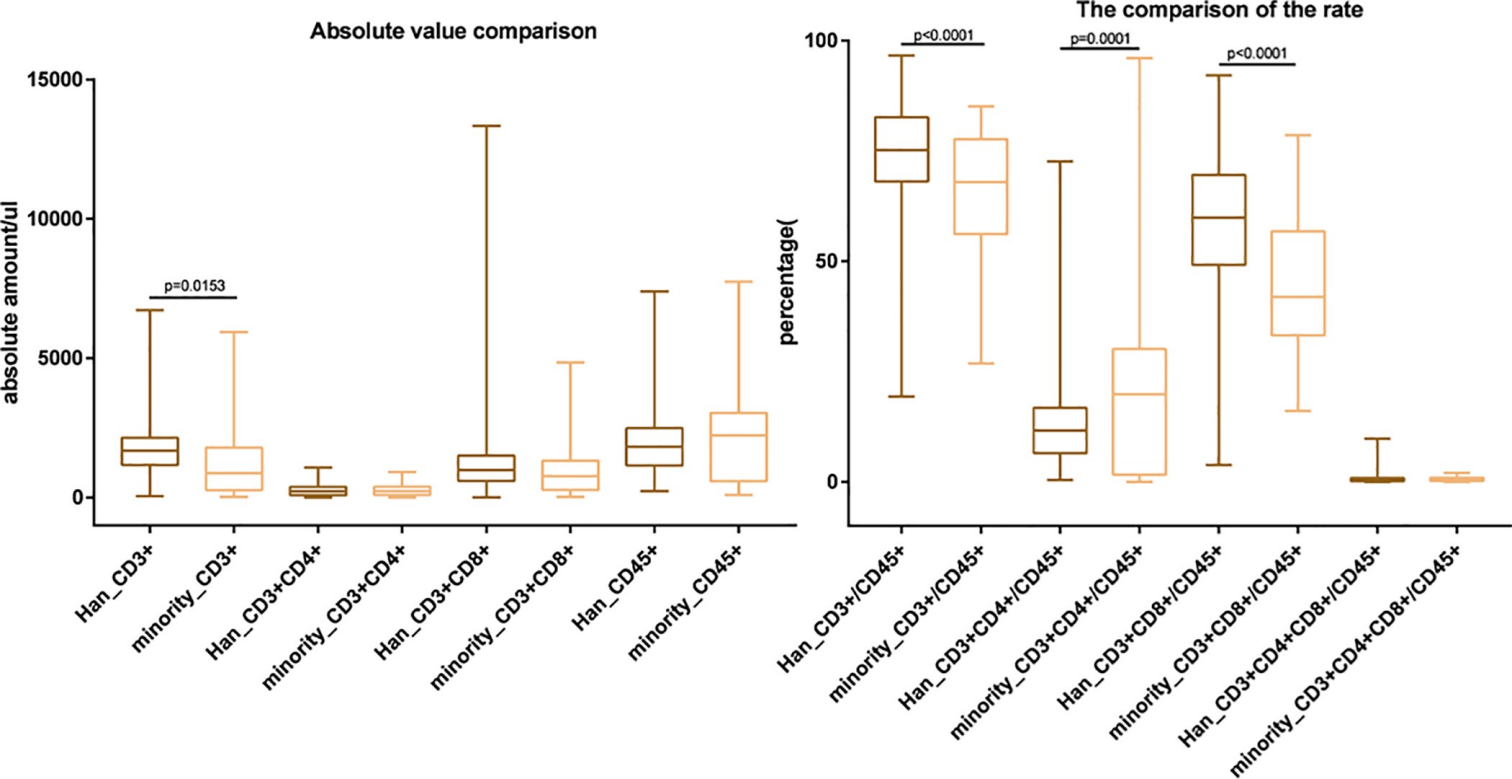
T lymphocyte subsets of Han and ethnic minority HIV+ patients. FACs data are presented as the mean ± SD for the comparison of CD3/CD4/CD8/CD45 subsets between Yunnan ethnic minorities.

In the context of HIV, ethnicity is associated with differences in CD4 T counts, the rate of CD4 decline, viral escape, and systemic immune activation [35,42–44]. As shown in Fig 4, the absolute number of CD3^+^ cells and the percentage of CD3^+^/CD45 and CD3^+^CD8^+^/CD45 cells were significantly higher in Han patients (1815 ± 987, 73.94 ± 11.61%, and 58.18 ± 15.42%) compared to ethnic minority patients (1417 ± 1130, 65.58 ± 14.55%, and 45.53 ± 16.02%) (P<0.001). The percentage of CD3^+^CD4^+^/CD45 cells was significantly higher in ethnic patients (19.67 ± 18.55%) compared to Han patients (13.04 ± 9.69%) (P<0.001). Although absolute numbers of Han patients had modestly higher numbers of CD3^+^CD8^+^, and minority patients had slightly higher numbers of CD3^+^, CD3^+^CD4^+^ and CD45^+^ T cells, the differences between the groups were not significant. These values may contribute to the hotspots of HIV-1 infection in Yunnan ethnic minorities including both Western and Northeast regions where the largest number of HIV sufferers occurs [45].

### T lymphocytes in patients of different ages

**Fig 5:**
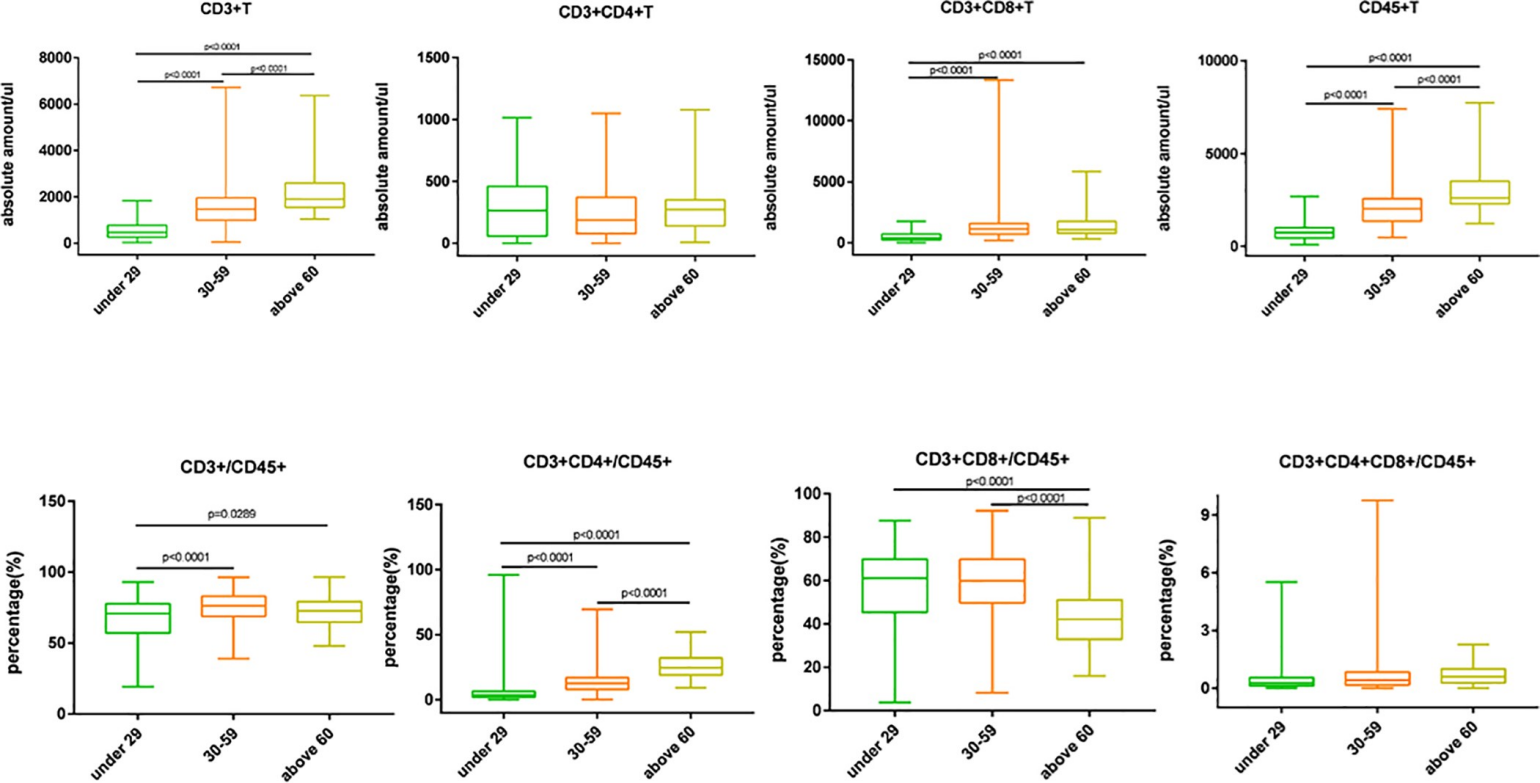
T lymphocyte subsets in newly diagnosed HIV/AIDS patients according to age. FACs data are presented as the mean ± SD for the comparison of CD3/CD4/CD8/CD45 subsets between different age groups.

HIV disproportionately afflicts young people both in China and worldwide. All newly diagnosed HIV/AIDS patients were divided into three groups based on age: ≤29, 30–59, or ≥60 years. Fig 5 summarizes the lymphocyte subsets present in each of these three age groups. Statistically significant differences in the absolute numbers of CD3^+^, CD3^+^CD8^+^, CD45^+^ and in the percentage of CD3^+^/CD45^+^, CD3^+^CD4^+^/CD45^+^, CD3^+^CD8^+^/CD45^+^T cells were found between the three age groups. The numbers of CD3^+^ T cells, CD3^+^CD8^+^ T cells, and CD45^+^ T cells were highest in the age ≥60 group. Significant differences were observed in the counts of CD3^+^, CD3^+^CD8^+^, and CD45^+^, and the ratio of CD3^+^/CD45^+^ to CD3^+^CD4^+^/CD45^+^ cells between the ≤29 and 30–59 groups. Other parameters decreased as age increased, however the differences did not show statistical significance.

The general linear model is composed of 7 factors: CD3+/CD45+, nationality, occupation, marital status, age, infection route, and degree of education. For the variable CD3+/CD45+, the influence of nationality and age showed a statistical significance(The P values were 0.01 and 0.025, respectively). In the general linear model of CD3+CD4+CD8+/CD45+, age and gender show an influence, and for the variable CD3+CD4+CD8+/CD45+, the influence of gender showed a statistical significance(The P values were 0.035), while age did not account for a significant influence(The P values were 0.198). These are shown in Table 3.

**Table 3:** Association between risk factors and T cells related index among newly diagnosed patients with HIV/AIDS.

	Coefficients (95%CI)	p-value
**CD3+/CD45+**	**R**^**2**^ **= 0.039**	**0.043**
**Nationality**	**-4.917(-8.66~-1.174)**	**0.01**
Occupation	-0.277(-0.751**~**0.197)	0.251
Marital Status	1.561(-1.304**~**4.426)	0.285
**Age Group**	**-3.218(-6.031~-0.406)**	**0.025**
Route of infection	-0.989(-3.321**~**1.343)	0.405
Degree of education	-0.321(-1.656**~**1.014)	0.637
**CD3+CD4+CD8+/CD45+**	**R**^**2**^ **= 0.019**	**0.040**
Age Group	0.084(-0.044**~**0.212)	0.198
**Gender **	**0.166(0.012~0.321)**	**0.035**

## Discussion

The Yunnan Province is located in Southwest China. Owing to its special location near the Golden Triangle, the HIV epidemic in this region has primarily spread through the use of injection drugs for many years [5–7]. It was previously reported that the mean number of CD4+ T cells in newly diagnosed HIV/AIDS patients in China in 2010 was 334 ± 302 cells/μl [20,33]. Amongst the 408 newly HIV-diagnosed patients in Yunnan, the baseline CD3+CD4+ T cell counts were 224 ± 191 cells/μl, which are significantly lower than those of Xiamen and Taizhou [46]. This suggests that HIV-infected patients in the Yunnan district are more likely to be diagnosed during the late stages of HIV infection (due to the progressive decline of CD4 numbers) compared to those in Xiamen and Taizhou. In addition, the lower CD4 counts may explain the greater risk of disease development and risk of progression to AIDS, reported to occur more rapidly in Yunnan compared to other regions [10,47–49].

In this study, it was found that the absolute numbers of CD3^+^, CD3^+^CD4, CD3^+^ CD8^+^, and CD45^+^T cells in infected women are higher than those in infected men. These biologically-based gender differences may arise from differential gene expression associated with the X chromosome [1]. Lower absolute numbers of HIV-specific CD8^+^ T-cells would be expected to lead to differential responses to ART therapy within the province, given the evidence for the significance of cytotoxic T-lymphocytes (CTLs) in the response to ART therapy [41].

Advanced age is also a strong predictor of accelerated HIV-1 progression and increased AIDS-related mortality [50–52]. The number of CD3^+^CD4^+^ T cells in patients aged ≤29 years old was higher than those aged > 30 years, suggesting that the number of T-lymphocytes decreases with increasing age. However, compared to patients aged 30–59, the group aged ≥ 60 showed a renewed increase in CD3^+^CD4^+^ T cells, suggesting that CD4^+^T cell damage was more chronic in patients aged 30–59 after HIV infection. Knowledge of these parameters is important in Yunnan given that the population of HIV-infected adults is progressively aging, due to more effective treatments that lower the viral load and increase life expectancy [50–52].

It was observed that CD3^+^ and CD3^+^/CD45 T cell percentages in the Han population were significantly higher than those of the ethnic minorities. HIV infects CD4^+^ T lymphocytes through binding of its viral envelope to cell surface expressed CD4 and to its co-receptors CCR5 or CXCR4 [53]. A 32 base pair deletion of the CCR5 gene has been shown to significantly reduce HIV entry and to disrupt the progression to AIDS [53–56]. CCR2-64I (A/A genotype) and SDF1-3'A are also known to protect against HIV infection, while CX3CR1 expression is associated with an increased risk of HIV-1 infection [57]. Mutations in CCR2 (CCR2-64I) also prolong the survival of HIV-infected individuals [54,55]. SDF1-3'A homozygotes show a protective effect against the onset of AIDS [55,58]. Chen and coworkers observed a high allele frequency of HIV-resistance genes CCR2b-64I and SDF1-3'A in the Han, Yi, and Hani ethnic populations [59]. In this study, the Yi and Hani ethnic groups accounted for 44% of the minority populations, and the T cell percentages were higher in these infected ethnic minority groups relative to the Han group. This suggests that differences in HIV-resistance genotypes may also be an important factor involved in the delay to HIV progression.

The proportion of patients with late stage diagnoses remains high in China, making effective treatments and viral remission difficult to achieve [33]. The frequency of opportunistic infections in HIV/AIDS patients increases as the CD3^+^CD4^+^ lymphocyte count decreases [23]. From the data, an estimated date of HIV infection was calculated for the diagnosed HIV-infected cohort based on previously-published methods [60]. Approximately 52% of newly diagnosed patients in this retrospective study were predicted to have been infected for more than 7 years at the time of diagnosis. These patients have reached a later disease-stage and have become highly susceptible to opportunistic infections. Approximately 24% of patients in the study were predicted to have been infected for 5–7 years, with the remaining 24% of patients estimated to have been infected for less than 5 years. Since late diagnosis greatly increases the risk of HIV exposure and spread, early diagnosis efforts and CD4 cell count testing should be priorities amongst high-risk populations within the Yunnan province. Regular assessment and monitoring of newly identified HIV-positive individuals should be accompanied by timely CD4 testing and rapid initiation of treatment. The National Free Antiretroviral Therapy Program (NFATP) in China has addressed the problem of poor access to ART therapy and has greatly reduced mortality among patients. Prompt CD4 testing is a critical step for successful HIV treatment, making it vital to reducing the rates of HIV/AIDS in this region.

One limitation of this study should be noted. The Han ethnic group accounted for the majority of study participants (349/408; 86%), and samples only consisted of infected individuals in the Kunming area, and are thus not fully representative of Yunnan. Sample selection bias and the small sample size may therefore influence our findings. Further studies comparing our findings to a range of ethnic groups should be conducted.

In conclusion, it is shown that low levels of CD4^+^ T lymphocytes in patients newly diagnosed with HIV/AIDS are found in the Yunnan province. Furthermore, T-cell subsets in these HIV patients were shown to be influenced by gender, age, and ethnicity which may contribute to disease progression. From this information, the most at-risk populations can be predicted and this can be used to inform HIV surveillance and management strategies. Older members of the Han nationality and men over 60, for example, may be more susceptible to HIV than younger Yi women. Of course, this requires prospective research and some actual prediction models, which can hopefully be conducted in future studies. Routine screening for HIV/AIDS in these populations can ensure timely and adequate clinical management in Yunnan, improving patient quality of life and reducing HIV morbidity.

## Supporting information

S1 File Description of methodological quality control and data rigor. This is a quality report on flow cytometry T lymphocyte subsets and it describe a technically sound piece of scientific research with data that supports the conclusions.
(DOCX)

S1 Dataset Original data. This is the source of all the data.
(XLSX)
