# Supplementary material for: AIDS prevention and control in the Yunnan region by T cell subset assessment
Source: PLoS One. 2019 Apr 18;14(4):e0214800. doi: 10.1371/journal.pone.0214800 (PMC6472762; doi:10.1371/journal.pone.0214800)

**Description of methodological quality control and data rigor**

1. The main flow cytometry analysis techniques used in the study were performed on a BD FACSCanto Ⅱ. The methodology has high precision, good accuracy, and the equipment is widely-used in industry.
2. Flow cytometry of T lymphocyte subsets were performed for one day of regular testing before indoor quality control was performed. Quality control can be detected during the testing of patient samples, and the test work established a perfect quality control system, which fully met the quality assurance guidelines for HIV and CD4+ T lymphocytes in patients with HIV. An example of quality control recorded in January 2018 was selected.

1. Our laboratory research project participated in an interlaboratory quality assessment organized by the clinical inspection center of the ministry of health every year. In China, the interlaboratory quality assessment activity organized by the ministry of health can be regarded as the most authoritative official agency for quality assessment. In the past three years, the results were excellent. The quality assessment results and certificates are as follows


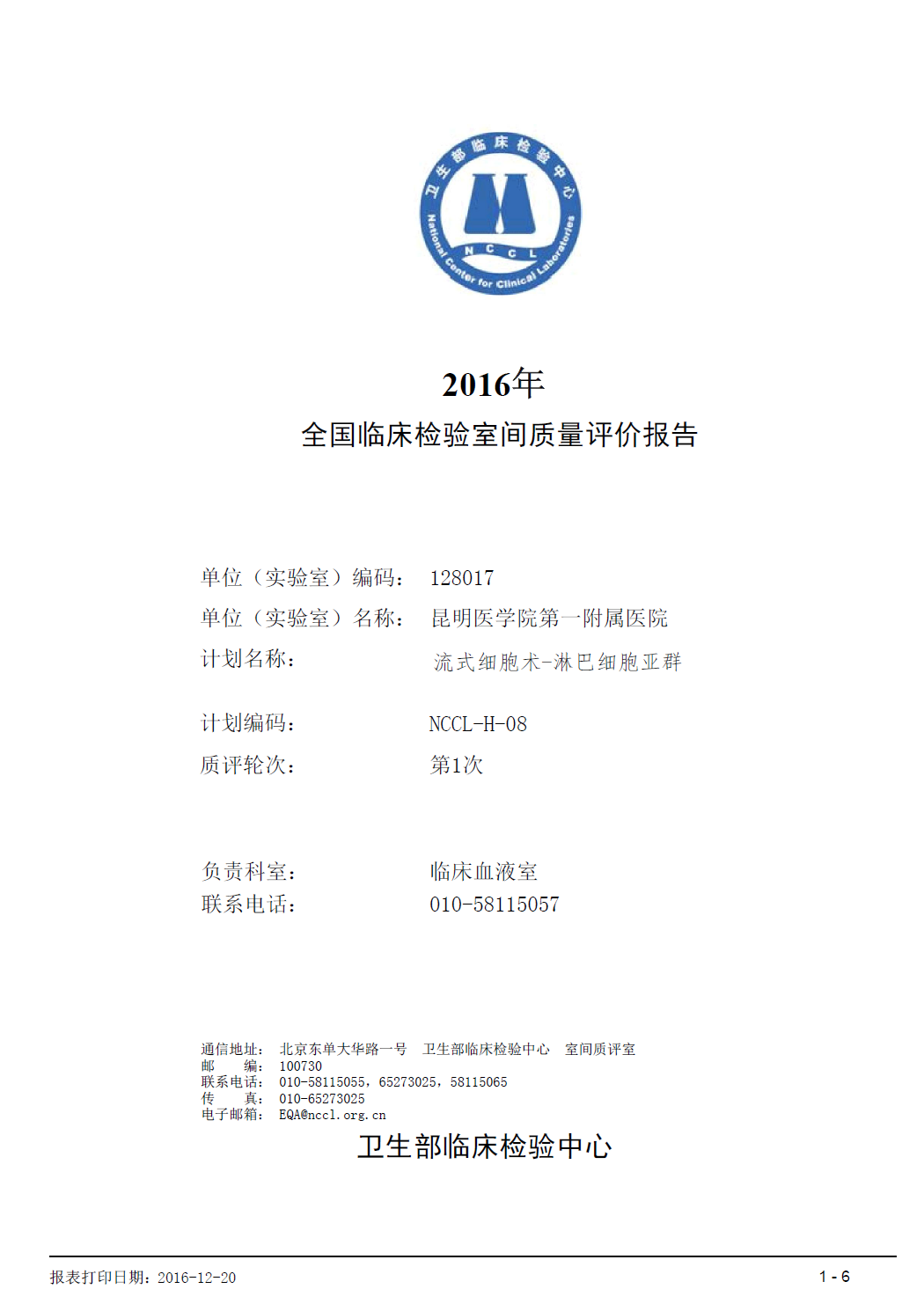


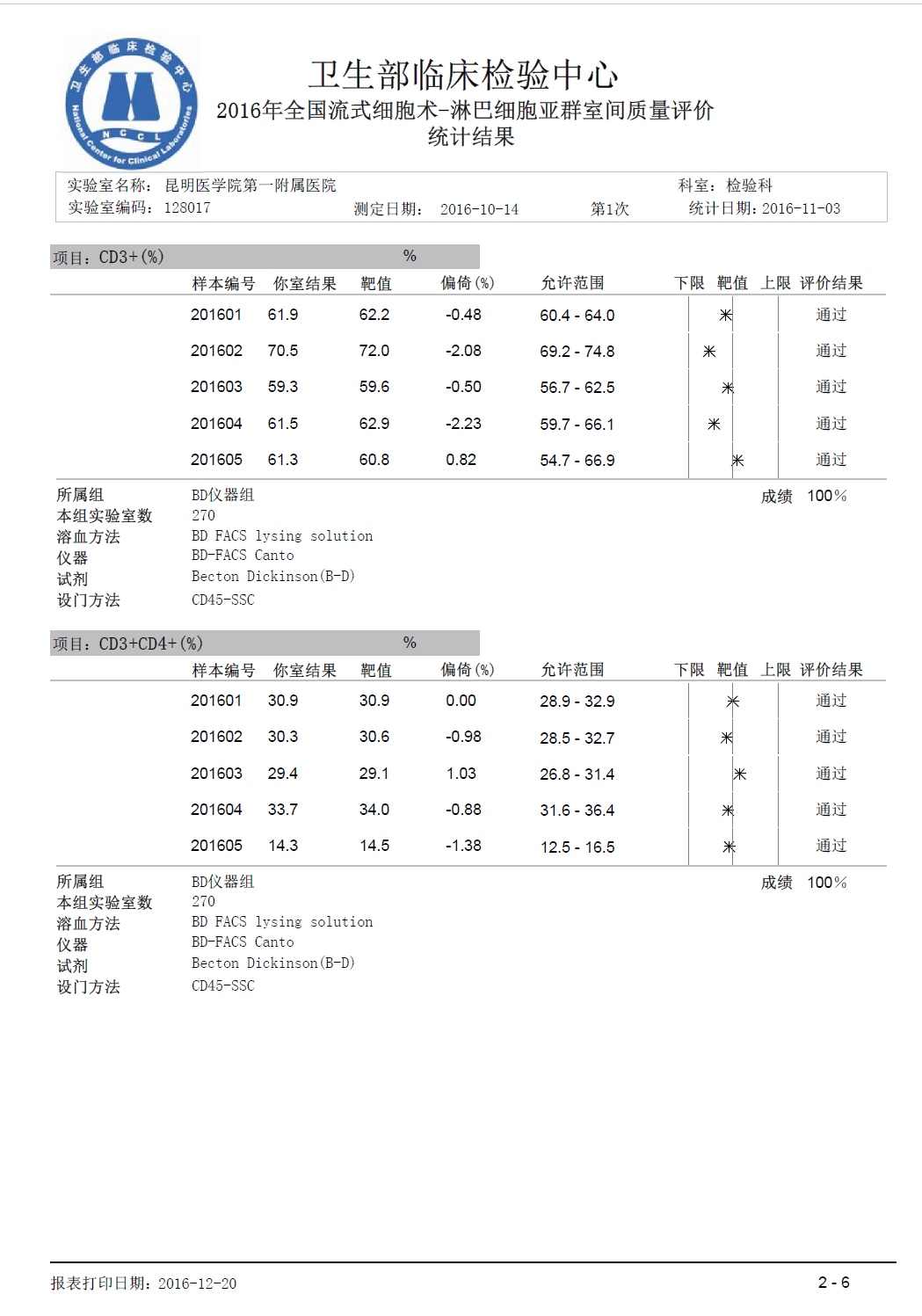


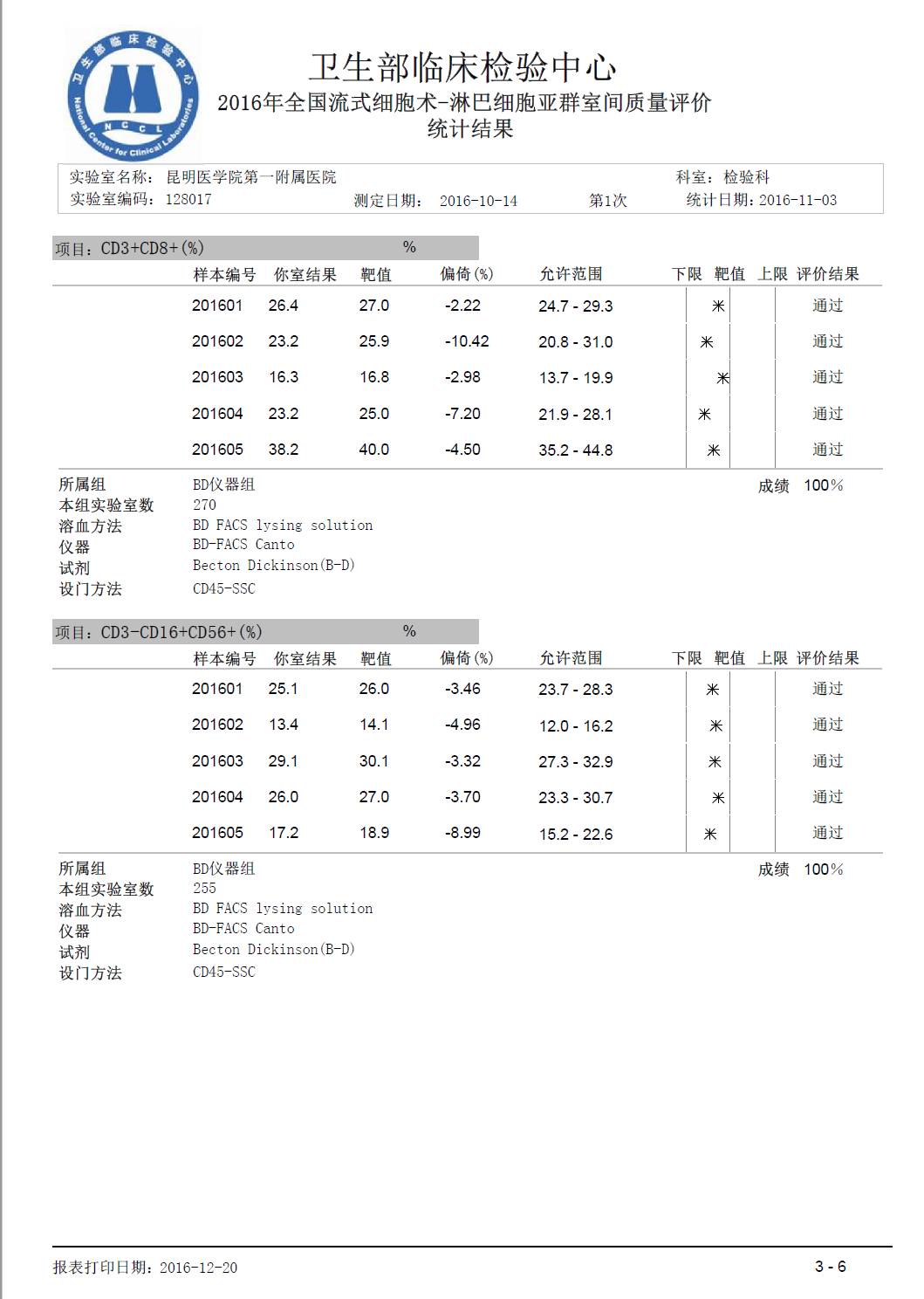


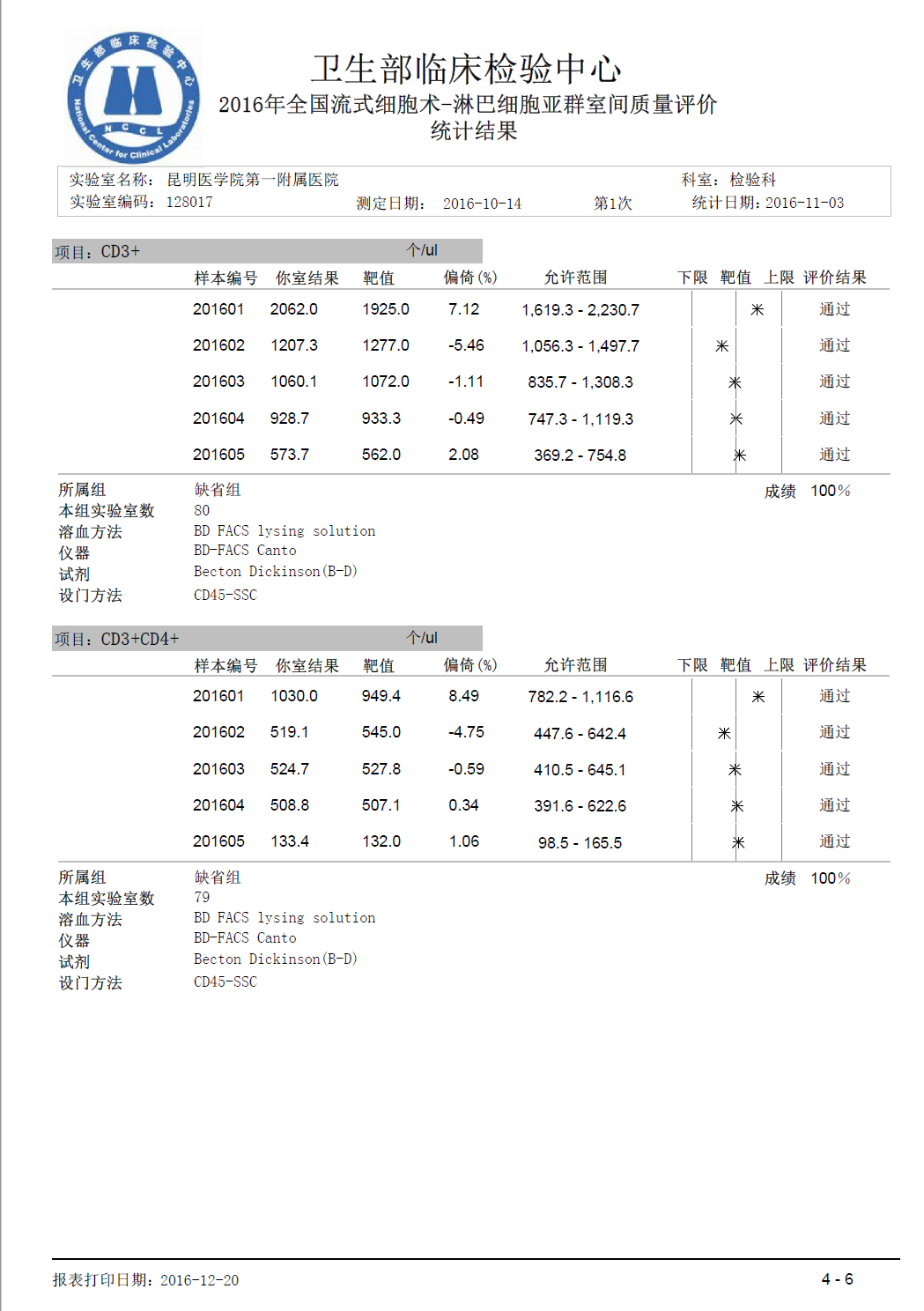


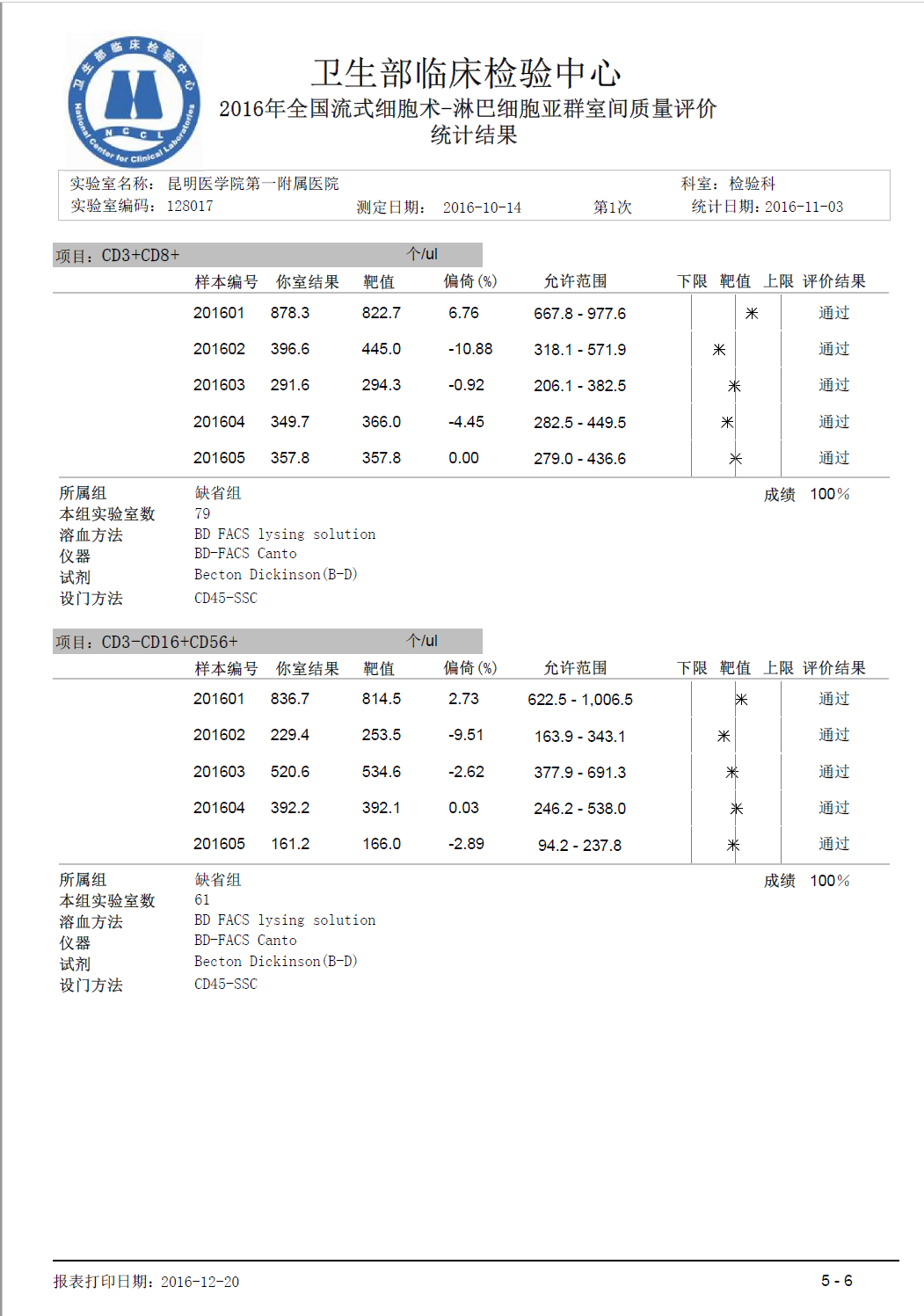


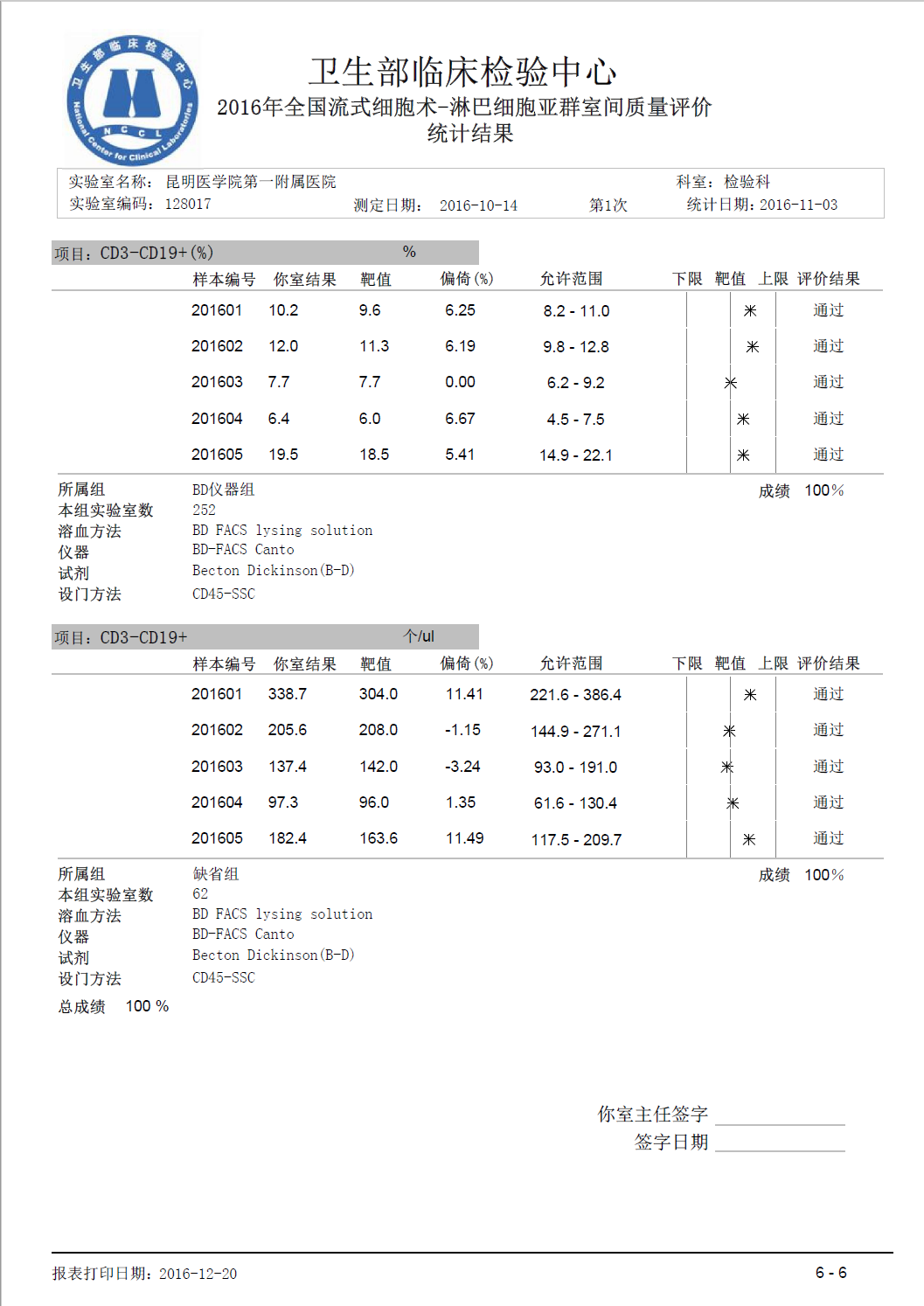


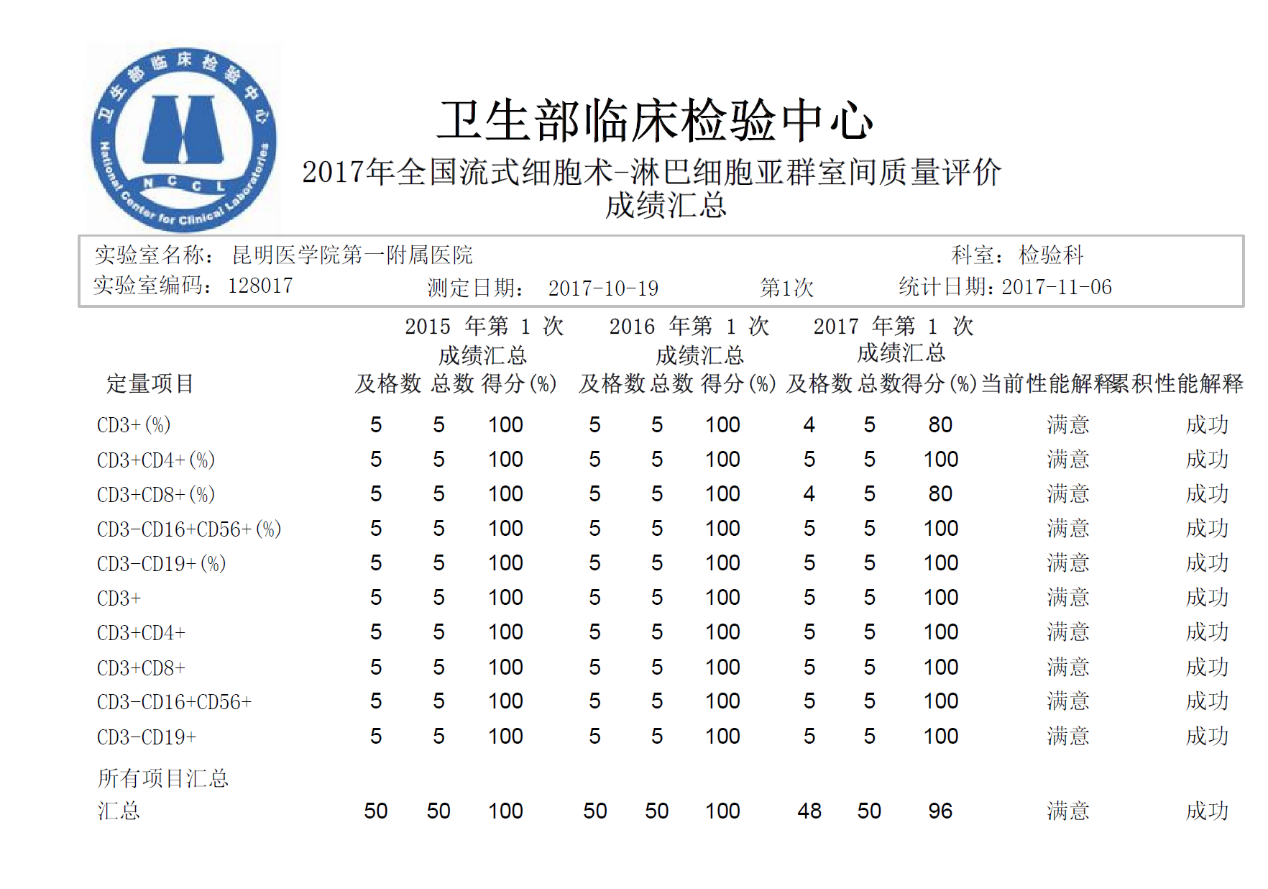


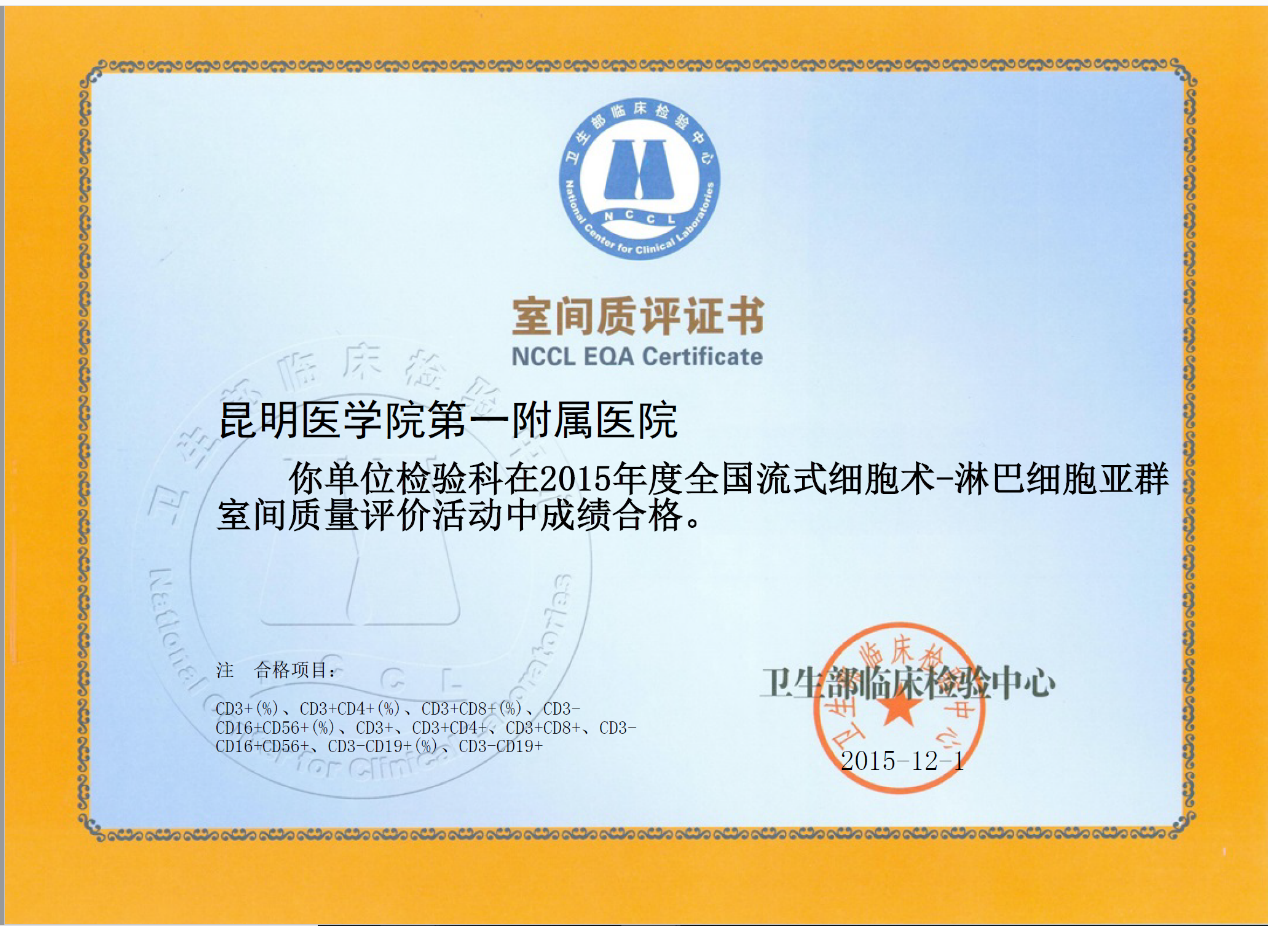


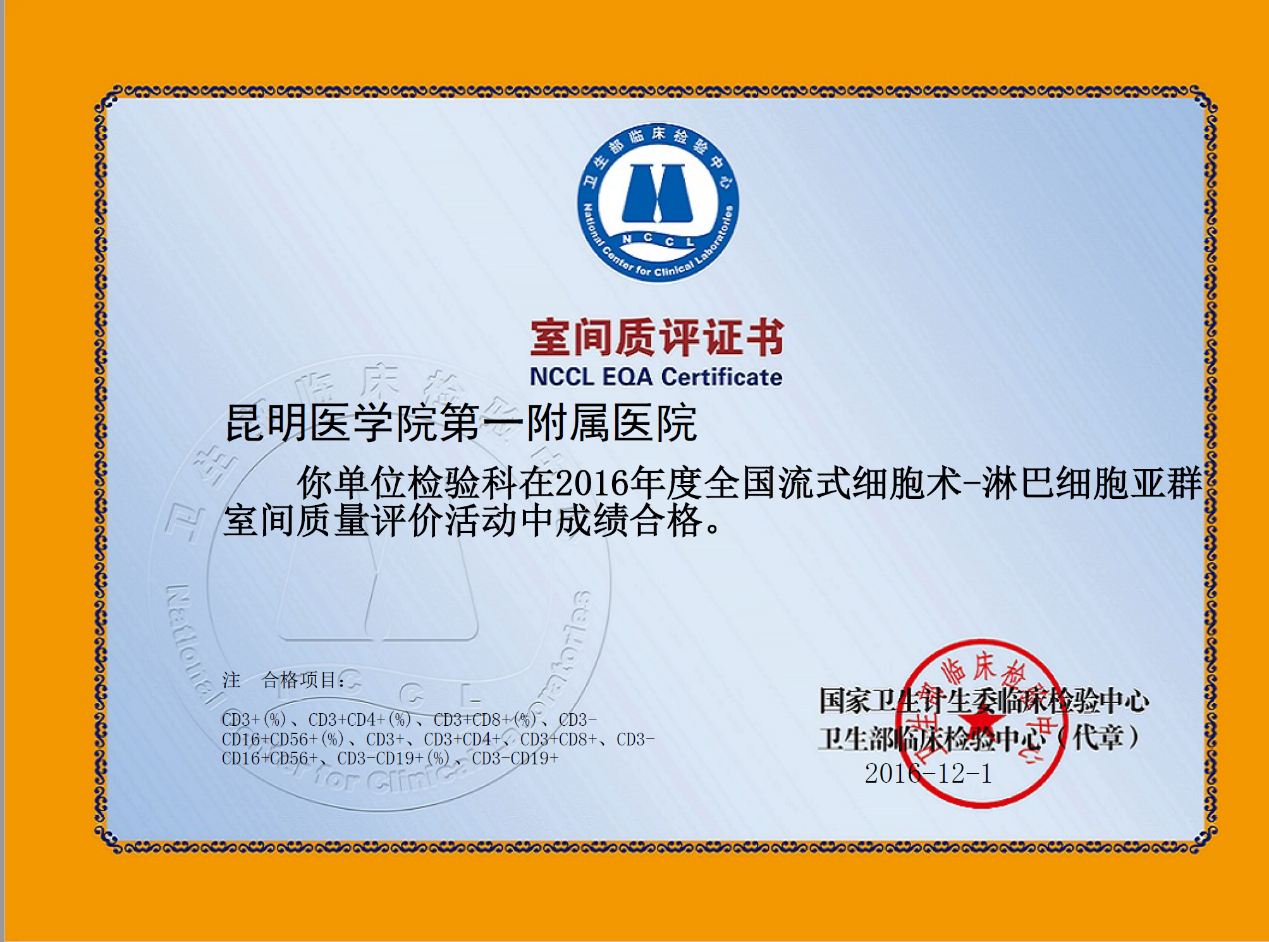


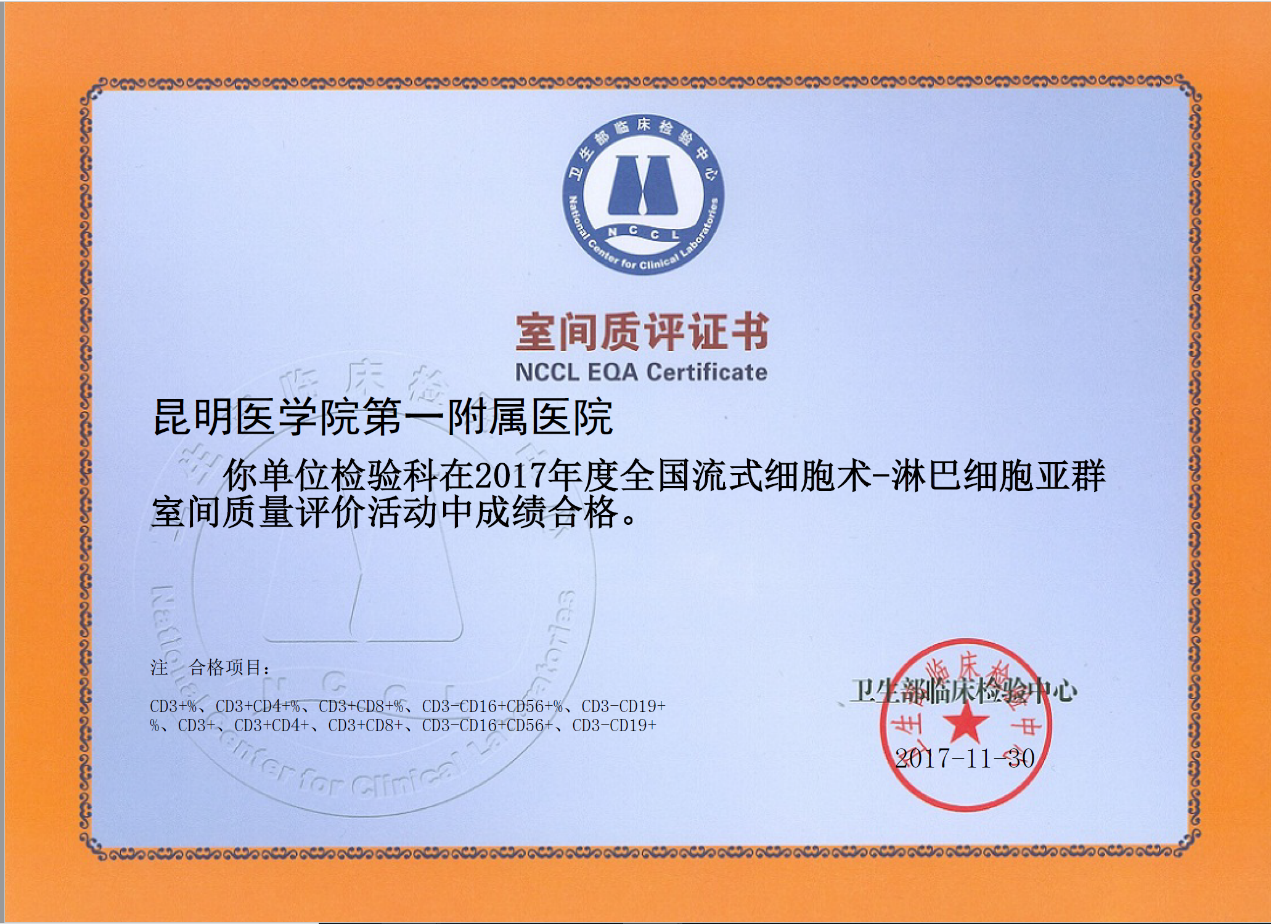


1. The first affiliated hospital of Kunming Medical University was approved by the National Accreditation Committee for Conformity Assessment of China in December 2012, and obtained ISO 15189, namely "accreditation criteria for quality and capacity of medical laboratories" (certificate attached). It also passed the third re-evaluation in 2018 and continued to maintain its accreditation. ISO 15189 is the general international standard. The European Union accreditation cooperation organization (EA) and the International Laboratory Accreditation Association (ILAC) accept ISO 15189 as the standard to recognize medical laboratories and have made it the main standard. After passing the ISO15189 accreditation, the test results of the first affiliated Kunming Medical University have received an 'international pass', and the test results are recognized by all laboratories that have passed ISO15189. The following is a certificate in English and a certificate in Chinese issued by the accreditation committee.


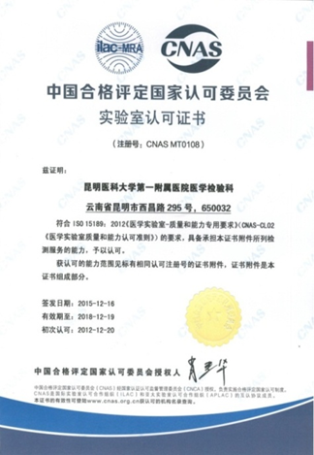


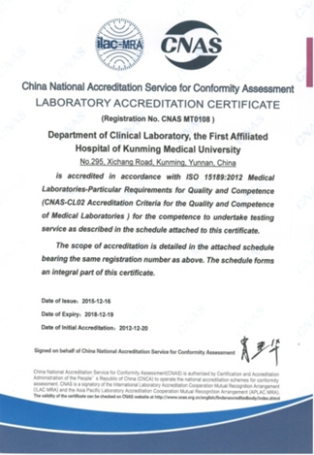

Supplement: S1 File — This is a quality report on flow cytometry T lymphocyte subsets and it describe a technically sound piece of scientific research with data that supports the conclusions. (DOCX) [file pone.0214800.s001.docx]
